# Multicriteria decision analysis (MCDA) in health care: a systematic review of the main characteristics and methodological steps

**DOI:** 10.1186/s12911-018-0663-1

**Published:** 2018-11-01

**Authors:** Talita D. C. Frazão, Deyse G. G. Camilo, Eric L. S. Cabral, Ricardo P. Souza

**Affiliations:** Departamento de Engenharia de Produção, Centro de Tecnologia, Universidade Federal do Rio Grande do Norte, Natal, 59072-970 Brazil

**Keywords:** Multicriteria decision analysis, Health care, Systematic review, Methodological aspects

## Abstract

**Background:**

The health area is one of the most affected systems on the perspective of decision-making with multiobjectives, thus becoming prone to errors in the final solution, however, multicriteria decision analysis (MDCA) appears as an aid tool for this process decision-making. Therefore,the present study aims to analyze and synthesize articles found in the literature, involing MCDA in health care, evaluating general issues and methodological aspects, structuring them in a single work.

**Methods:**

Surveys in the bibliographic databases SCOPUS and PUBMED indicated 1852 documents on the subject, however after a careful verificatios, 66 studies were selected to be analyzed completely. The data extracted from the included articles were organized into a spreadsheet for the preparation of analysis, and the technique used was descriptive statistics.

**Results:**

It was possible to identify a growth trend in the application of the MCDA in the health area, but no dominance was identified in relation to the authors of the publication and the periodicals where they are published, but some countries stood out in terms of the number of published researches, such as: Canada and Turkey. In defining the decision problem, and in defining criteria, the “literature” presented the greatest demand for those who wish to structure their decision problem. Finally, it was verified by the analysis of the problem, that the MCDA to solve the problems of ranking has comprehensive application and that there is a greater incidence in the use of the AHP and Logic methods Fuzzy.

**Conclusion:**

With this, it is possible to observe, through the data of this review, that more than the multicriteria methods, the multicriteria decision model has been highlighted, also in the health area. In addition, the study can guide new applications and techniques using MCDA in the health care.

## Background

The issue of multiple objectives is always present in the problems within organizations; Increasing the complexity of decisions. In this setting, it is necessary to find techniques that include in the decision-making process, the greatest number of criteria that guide and influence decisions, in order to reduce errors. However, most of the time this procedure is not easy to perform, since in many situations, the criteria for decision making are conflicting, increasing the level of uncertainty of the final response [1–3].

In order to increase the reliability and credibility of the chosen solution, decision support methodologies, such as Multicriteria Decision Support Methods (MCDA), have emerged [4, 5]. These methods are intended to assist in the decision-making process, in order to minimize the responsibility of the final decision-maker, and to guarantee a solution in accordance with the criteria in question [6].

In the health area, these procedures are even more complex, since they involve not only technical or economic issues, but also the human factor, causing conflicts of interest and hindering the final decision [2]. Therefore, many studies, using MCDA, are carried out with the aim of optimizing health systems as a whole [7–10].

Some studies have been concerned with analyzing a specific application sector, such as the evaluation of health technology [11]. Others depart for a more humane view, evaluating studies aimed at assessing patient preference [12]. And there are those who go further, and aim to know and analyze the MCDA in a complete way in health [13, 14].

As there are a great number of studies involving MCDA in the health area, this study aims to analyze and synthesize the information found in the literature, by evaluating general questions and methodological aspects, structuring in a single work the main articles.

For this, it was developed in a systematic revision model, which is subdivided into two stages of evaluation. First the analysis of the general questions of the article, aiming to know and evaluate the scenario of the MCDA studies in the health care. The second stage will be the structural analysis of the research.

## Conceptualization of the MCDA

There is a multicriteria decision problem, when Decision Maker (DM) faces a situation with at least two alternatives of action with conflicting objectives among which it must choose [15, 16]. These decisions are rarely made by a single individual, even if the responsibility for the decision rests on a well-identified DM, the decision will usually be the product of an interaction between that individual’s preferences and those of other actors or stakeholders [17]. In addition to DM, there may still be: the Analyst (provides methodological support for the decision process); the Client (an intermediary between the DM and the Analyst) and the Specialist (a professional who knows the mechanisms of behavior of the object of study) [17].

The construction of models and the choice of methods are directly linked to the actors of the decision-making process. The meaning of the decision-making expressions and decision support method may vary in the literature [18, 19]. In the present research, it is considered that a multicriteria decision model is a formal representation and with simplification of the decision problem with multiple objectives faced by the DM, already a method of support the multicriteria decision is a methodological formulation or a theory, with axiomatic structure well-defined, which can be used to construct a decision model [15].

In problems that use the multicriteria decision model, there is a need to obtain the alternatives, criteria, weights, decision matrix and scale. According to Dolan [20], Baltussen et al. [21], Belton and Stewart [22]: An alternative is a course of action assessed through a decision-making process; The criteria are the performance measures, by which, the options will be judged and carefully selected to ensure integrity, viability and mutual independence, avoiding redundancy and an excessive number of criteria; As for weight, it is a number that expresses the relative importance of the criteria against which alternatives are compared; a decision matrix is a table that presents the performance of each alternative according to the criteria and is measured at appropriate scales; The decision matrix is used inversely with the terms performance matrix; Finally, the term scale refers to an instrument, in which the performance of an alternative is measured. Two types of data can be measured on these scales, in particular, qualitative and quantitative scales.

### Stages of the multicriteria decision model

For this study, it was considered the structure proposed by Diaby and Goeree [23], which consists of three stages: (I) Define the limits of the problem - The study must have a well elaborated and explicit objective, where a central problem must be pre-definined, thus serving as a guide to the study itself; (II) Identify the evaluation criteria - The next step is to select the criteria for the analysis of the research, ie, which criteria will be used to evaluate the problem under study; (III) Select a multicriteria model - After completing the application methodology, one must select the model to be used in the study, this model must be selected considering the problem defined in step I.

Considering that, the problem has been defined clearly and the criteria to assist in decision-making are pointed out, then a multi-criteria evaluation model can be chosen in order to meet the conditions and needs of the problem of interest [20]. Roy [17] addresses four types of problems: (I) Choice - it is the selection of a subset as small as possible, so that a single action can eventually be chosen; (II) Sorting - is the classification that leads to an assignment of each action to a category, where categories are defined as an advantage according to certain norms that deal with the final destination of the actions that will be assigned to them; (III) Ranking - is a ordination, which is obtained by placing all actions, or simply the “most attractive ones”, in equivalences that are totally or partially ordered according to preferences; (IV) Description - is the development of a description of actions and their consequences in appropriate terms.

Diaby and Goeree [23] further conclude that for each type of problem, there are appropriate MCDA methods.

It is of interest of this research only the three problematic issues, because the main objective is accomplished in the analysis of articles, which present the resolution of problems through a choice, a sorting procedure, or still resulting in a rank of the evaluated alternatives.

## Methods

### General search data

The review of the literature presented in this study is based on bibliographic databases SCOPUS and PUBMED, including MEDLINE, PMC (PubMed Central) e NCBI Bookshelf, which were searched in March 2017. No language, publication date, or publication status restrictions were imposed to reach as many articles as possible. The consideration of two data repositories seeks to avoid a possible bias and/or omission in the final set of selected articles. To control the quality of published works, research was limited to journals.

The survey included articles that have MCDA application in health care. Thus, it is possible to investigate how and for what purpose the researchers and practitioners use MCDA to aid decision making in health care.

**Fig. 1:**
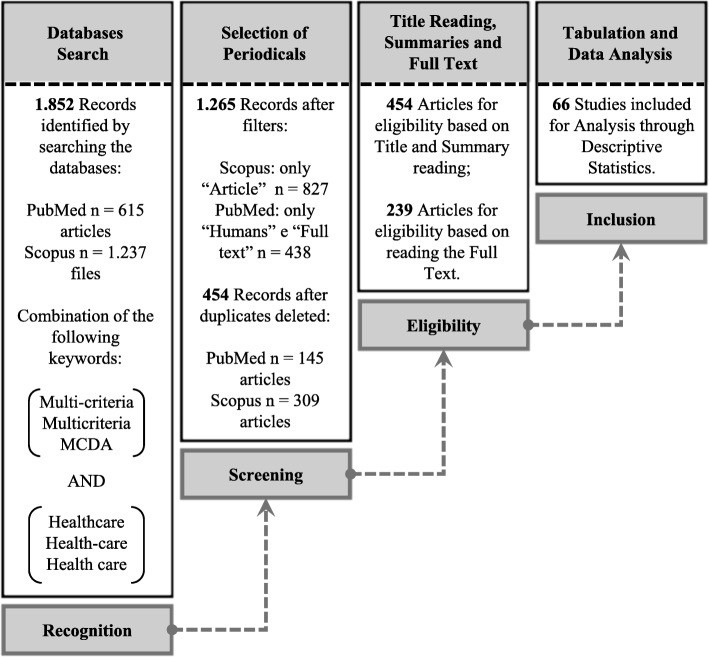
Sequential steps which were followed for the collection and analysis of the data of the included articles

In the SCOPUS repository the searches were given for “title, abstract and keywords”, the added filters are presented in Fig. 1. The data extracted from the articles selected were: Author; Date of the survey; Location (Country where the survey was conducted, if not reported the country of origin of the main author); Periodical of publication; Title of study; Type of intervention (according to its characteristics were classified into six classes); Type of problem (choice, ranking and sorting). How did the problem arise? (How happened definition of the problem?) (According to their characteristics were classified into five classes); How did the definition of the criteria occur? (According to their characteristics were classified into five classes); and what is the weighting method used?

Table 1 summarizes the main features of the studies included in the review.

**Table 1:** Summary of main features of included studies

Author	Journal	Country	Intervention type	Problem type	Problem definition	Definition of criteria	Method
Tarimcilar and Khaksari [50]	Socioecon. Plann. Sci.	USA	Management	Ranking	Decision-makers	Decision-makers	AHP
Sinuany-stern et al. [64]	Locat. Sci.	Israel	Management	Choice	Group discussion	Group discussion	AHP
Dolan [48]	Heal. Expect.	USA	Health Care	Choice	Group discussion and specialists	Literature	AHP
Baltussen and Niessen [21]	Cost Eff. Resour. Alloc.	Netherlands	Resources	Sorting	Decision-makers	Decision-makers	WHO-CHOICE
Singh et al. [33]	BMC Med. Inform. Decis. Mak.	USA	Management	Sorting	Decision-makers	Decision-makers	AHP
Ágnes [66]	Magy. Onkol.	Hungary	Resources	Ranking	Literature	Literature	FUZZY LOGIC
Doerner et al. [27]	Eur. J. Oper. Res.	Senegal	Location	Choice	Literature	Literature	P-ACO
Goetghebeur et al. [25]	BMC Health Serv. Res.	Canada	Management	Sorting	Literature	Literature	EVIDEM
Jehu-appiah et al. [61]	Value Heal.	Ghana	Health Care	Ranking	Decision-makers	Interview	WHO-CHOICE
Kuzma et al. [49]	Risk Anal.	USA	Management	Sorting	Literature	Group discussion	IOA
Sustersic et al. [32]	J Int Med Res	Slovenia	Health Care	Sorting	Specialists	Literature	HMADM
Dursun et al. [31]	World Acad. Sci. Eng. Technol.	Turkey	Pollution	Ranking	Group discussion and specialists	Group discussion	FUZZY LOGIC
Goetghebeur et al. [39]	Cost Eff. Resour. Alloc.	Canada	Health Care	Sorting	Literature	specialists	EVIDEM
Danner et al. [67]	Int. J. Technol. Assess. Health Care	Germany	Health Care	Ranking	Decision-makers	Group discussion	AHP
Dursun et al. [42]	World Acad. Sci. Eng. Technol.	Turkey	Pollution	Ranking	Group discussion and specialists	Literature	FUZZY LOGIC
Dursun et al. [43]	Resour. Conserv. Recycl.	Turkey	Pollution	Choice	Literature and specialists	Literature and specialists	FUZZY LOGIC
Lee and Kwak [68]	J. Med. Syst.	South Korea	Management	Ranking	Group discussion	Group discussion	AHP
Padma and Balasubramanie [35]	Expert Syst. Appl.	India	Health Care	Ranking	Specialists	Literature	FUZZY LOGIC
Tony et al. [40]	BMC Health Serv. Res.	Canada	Health Care	Choice	Decision-makers	Literature	EVIDEM
Defechereux et al. [62]	BMC Health Serv. Res.	Norway	Others	Sorting	Literature	Specialists	FUZZY LOGIC
Mirelman et al. [63]	Value Heal.	Brazil, Nepal, Norway, Uganda and Cuba	Others	Ranking	Literature	Specialists	FUZZY LOGIC
Hummel et al. [56]	Appl. Health Econ. Health Policy	Netherlands	Health Care	Choice	Decision-makers	Literature	AHP
Isoke and Van Dijk [69]	Water Environ. J.	Uganda	Pollution	Sorting	specialists	specialists	AHP
Liu et al. [10]	Waste Manag.	China	Pollution	Ranking	Literature	Specialists	VIKOR
Lu et al. [26]	Decis. Support Syst.	Taiwan	Resources	Choice	specialists	Literature	VIKOR
Özkan [45]	Waste Manag. Res.	Turkey	Pollution	Ranking	Literature	Literature	ANP and ELECTRE III
Diaz-Ledezma et al. [47]	Clin. Orthop. Relat. Res.	USA	Health Care	Ranking	Literature	Literature	AHP
Liu et al. [51]	Waste Manag.	China	Pollution	Choice	Literature	Specialists	ITL-MULTIMOORA
Oddershede et al. [70]	Int. J. Comput. Commun. Control	Chile	Resources	Ranking	Decision-makers	Literature	OPENET
Reddy et al. [34]	Public Health	United Kingdom	Health Care	Ranking	Group discussion and specialists	Group discussion	AHP
Stromme et al. [71]	Dev. World Bioeth.	Norway	Health Care	Ranking	Literature	Literature	FUZZY LOGIC
Til et al. [57]	Cost Eff. Resour. Alloc.	Netherlands	Resources	Ranking	Literature	Specialists	EVIDEM
Venhorst et al. [58]	Cost Eff. Resour. Alloc.	Netherlands	Health Care	Sorting	Group discussion	Specialists	DELPHI
Ahmadi et al. [65]	Int. J. Med. Inform.	Malaysia	Resources	Sorting	Literature	Specialists	ANP
Cabrera-Barona et al. [72]	IInt. J. Health Geogr.	Ecuador	Others	Sorting	Literature	Literature	AHP
Dehe and Bamford [9]	Expert Syst. Appl.	United Kingdom	Location	Choice	Literature	Decision-makers	AHP
Diaby and Lachaine [38]	Appl. Health Econ. Health Policy	Canada	Management	Ranking	Decision-makers	Decision-makers	WHO
Graaf et al. [55]	Biomed Res. Int.	Netherlands	Health Care	Ranking	Group discussion and specialists	Group discussion and literature	SMAA-O
Kulak et al. [44]	Appl. Soft Comput.	Turkey	Resources	Choice	Group discussion	Group discussion	FUZZY LOGIC
Kuruoglu et al. [73]	BMC Med. Inform. Decis. Mak.	Peru	Others	Choice	Group discussion	Literature and specialists	AHP
Paolucci et al. [53]	Health Policy Plan.	China	Health Care	Ranking	Decision-makers	Decision-makers	DCE
Ritrovato et al. [74]	Value in Health	Italy	Health Care	Choice	Literature	Literature	AHP
Wahlster et al. [75]	Heal. Res. Policy Syst.	Germany	Others	Sorting	Literature	Specialists	EVIDEM
Carnero [30]	Shock Vib.	Portugal	Resources	Choice	Literature	Literature	FAHP
Carnero and Gómez [76]	BMC Med. Inform. Decis. Mak.	Spain	Resources	Choice	Literature	Group discussion and literature	MACBETH
Delice and Zegerek [8]	Appl. Math. Inf. Sci.	Turkey	Turkey	Ranking	Ranking	Specialists	FUZZY LOGIC- GRA
Diaby et al. [46]	Expert Rev. Pharmacoecon. Outcomes Res.	USA	Resources	Choice	Group discussion	Group discussion	ELICIT
Gómez and Carnero [77]	Int. Fed. Autom. Control	Portugal	Resources	Choice	Literature	Group discussion	MACBETH
Hongoh et al. [29]	Int. J. Environ. Res. Public Health	Canada	Health Care	Ranking	Group discussion and decision-makers	Group discussion and literature	PROMETHEE II
Hussain and Malik [36]	J. Health Organ. Manag.	Abu Dhabi	Pollution	Ranking	Literature	Literature	AHP
Hussain et al. [78]	Bus. Process Manag. J.	United Arab Emirates	Health Care	Choice	Specialists	Literature and specialists	AHP
Kalhor et al. [79]	J. Biol. Today’s World	Iran	Pollution	Choice	Specialists	Specialists	TOPSIS
Kim and Kim [80]	Technol. Forecast. Soc. Chang.	Korea	Health Care	Ranking	Specialists	Specialists	AHP
Lu et al. [52]	Int. J. Environ. Res. Public Health	China	Pollution	Choice	Literature	Specialists	ITI-TOPSIS
Mahfoud et al. [81]	Am. J. Appl. Sci.	Morocco	Resources	Ranking	Specialists	Specialists	PROMETHEE
Merola et al. [82]	Int. J. Bus. Syst. Res.	Italy	Resources	Ranking	Group discussion and literature	Literature	AHP
Mohamadi et al. [83]	Shiraz E-Med J	Iran	Health Care	Choice	Literature and specialists	Group discussion and literature	SAW
Nilashi et al. [84]	Technol. Forecast. Soc. Chang.	Malaysia	Resources	Sorting	Literature	Literature	ANP
Rebolledo et al. [59]	J. Environ. Manage.	Spain	Pollution	Sorting	Literature	Literature	AHP
Shafii et al. [85]	Osong Public Heal. Res. Perspect.	Iran	Others l	Ranking	Literature	Literature	TOPSIS
Wagner et al. [41]	PharmacoEconomics	Canada	Health Care	Ranking	Decision-makers	Literature	EVIDEM
Wang et al. [54]	Comput. Ind. Eng.	China	Others	Ranking	Group discussion and decision-makers	Literature and specialists	IVIF-COPRAS
Carnero and Gómez [28]	Sustain.	Spain	Resources	Choice	Literature	Group discussion	MACBETH
Hancerliogullari et al. [86]	BMC Med. Inform. Decis. Mak.	United Kingdom	Resources	Ranking	Decision-makers	Specialists	TOPSIS
Hillerman et al. [87]	J. Comput. Sci.	Brazil	Management	Ranking	Group discussion and literature	Literature	AHP
Hillerman et al. [87]	J. Comput. Sci.	Brazil	Management	Ranking	Group discussion and literature	Literature	AHP
Wagner et al. [60]	BMC Cancer	France, Italy and Spain	Health Care	Ranking	Literature	Literature	FUZZY LOGIC

### Search steps and search criteria

The study was performed in four stages, as shown in Fig. 1, obeying some inclusion and exclusion criteria, as shown in Table 2. The research was performed independently in an unblinded standardized manner by two reviewers. Agreements between reviewers were resolved by consensus. We developed a data extraction sheet (based on the what we want to extract from the articles). The articles were divided between two authors for data extraction. Already, disagreements were resolved by discussion between two review authors; if no agreement could be reached, it a third author would decide. The steps and their respective criteria are described below:

**Table 2:** Criteria used for inclusion and exclusion of the studies in the review

**Inclusion criteria**	
** Inclusion criteria for title and abstract**	**Inclusion criteria for full text**
Is a health intervention aided by the MCDA.	The MCDA is structured according to the steps proposed by Diaby and Goeree (2014); Provide the necessary information for analysis of general data and methodological steps.
**Exclusion criteria**	
**Exclusion criteria for title and abstract**	**Full text exclusion criteria**
Does not present abstract; Does not present the full text available; Be a review article; Does not be an MCDA application and / nor Does; Not be MCDA application in the health area.	Be an MCDA application, however: Does not follow the steps outlined above; Does not make clear the structuring of the problem; Does not make clear the criteria and their origin; Does not present decision matrix; Does not present the quantitative weighting of the criteria; Presents a purely mathematical model.

**(I) Identification** Nine words combinations were searched for in title, abstract and keywords fields. This words were: Multi-criteria and Healthcare; Multi-criteria and Health-care; Multi-criteria and Health care; Multicriteria and Healthcare; Multicriteria and Health-care; Multicriteria and Health care; MCDA and Health-care; MCDA and Healthcare; MCDA and Health care. The use of these combinations is justified by the need to restrict the search of articles relevant to research, and because there is a variation of the terms in the literature. Figure 1 shows the amount of documents resulting after the execution of each of the procedures.

**(II) Screening** For the first selection of studies, filters were applied and duplicate documents removed;

**(III) Eligibility** After the elimination of duplicate articles, a title, abstract and full text were read. This stage of eligibility consisted of two phases. In the first, the titles and abstracts were read, obeying an inclusion criterion (Table 2). If they answered the prerequisite, the full text would be downloaded. For the second phase, which was the reading of the articles, pre-selected and downloaded, two inclusion criteria and seven exclusion criteria were determined (Table 2);

**(IV) Inclusion** The data extracted from the included articles were organized into a spreadsheet for the preparation of analysis, and the technique used was descriptive statistics. The results are presented and discussed in “Results” section.

## Results

### Analysis of the general aspects

Amount of 1852 publications were identified in the databases by the combination of keywords. After a refined search (Scopus: only, “Article” *n*=827, PubMed: only, “Humans” e “Full text” *n*=438) and after adjusting for duplicates 454 remained. Of these, 239 studies were discarded because after reviewing the abstracts it appeared that these papers clearly did not meet the criteria. The full text of the remaining 239 articles was examined in more detail. It appeared that 173 studies did not meet the inclusion criteria as described (Fig. 7). Sixty-six studies met the inclusion criteria and were included in the review (See flow diagram Fig. 1).

**Fig. 2:**
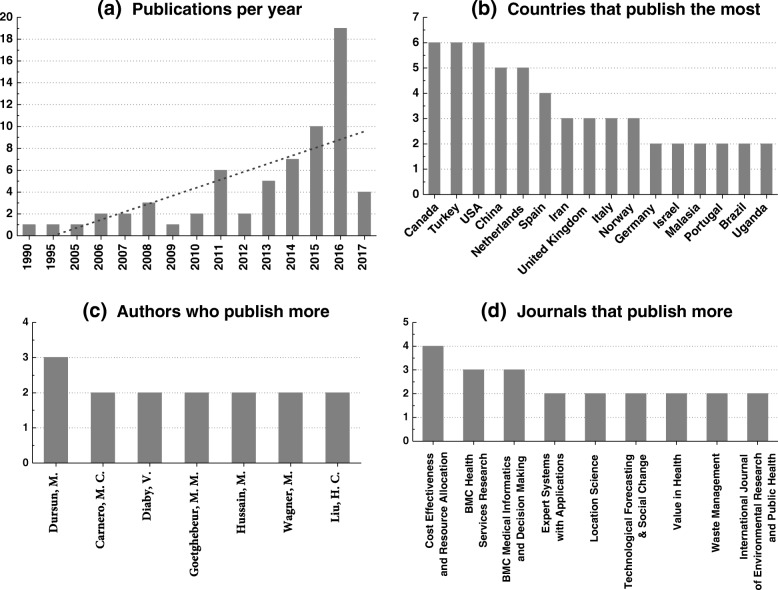
Four chart which show the analysis of the general aspects of the articles included, such as: **a** demonstration of publications, **b** main countries, **c** authors, and **d** journals

Within the line of general analysis of the studies selected for analysis, it is sought to identify the line of growth or decrease of research in the area, in addition to diagnosing the main authors and journals, and the countries that publish the most over time. The analyzed data are shown in Fig. 2.

Figure 2a shows the number of subject publications per year. It is evidenced a growth of publications on the MCDA theme in health, with great emphasis from the year 2014.

Figure 2b addresses the location of study application and / or origin of the main authors of the articles selected in the study. We took into account the inclusion criteria for full text (see Table 2). Thus the countries Canada, Turkey and the USA lead in quantity of publications on the subject, each with 6 articles. In this way, we can affirm that their studies supported the structured methodology of the MCDA, which were the focus of this research.

Throughout the review, thirty-three countries were surveyed (Table 1), however, only those who had more than one total publication were included in the analysis.

Figure 2c demonstrates the authors who stand out most in the theme. We can visualize the seven authors who had more than one publication among the analyzed studies, and it is possible to say in front of the graph that there is no author who stands out from the others, since Dursun, with three publications in all, is followed by plus six authors with two publications each.

According to Fig. 2d, brings the most published papers on the topic of MCDA in the health area. Eight newspapers stand out, with Cost Effectiveness and Resource Allocation being more prominent with four publications.

Finally, a parameter widely used in the literature to identify and classify the most important works is the number of times it is cited [24]. In Table 3 are presented the twenty most cited papers in relation to 66 selected ones, as well as the number of citations registered in the Scopus database, in June 2018.

**Table 3:** Twenty most cited papers among the 66 selected papers

Research	Year of publication	Scopus citations
Baltussen et al. [21]	2006	265
Goetghebeur et al. [25]	2008	76
Goetghebeur et al. [39]	2010	70
Lu et al. [26]	2013	64
Doerner et al. [27]	2007	63
Dursun et al. [31]	2010	49
Jehu-Appiah et al. [61]	2008	47
Liu et al. [10]	2013	43
Tony et al. [40]	2011	41
Singh et al. [33]	2006	34
Liu et al. [51]	2014	34
Defechereux et al. [62]	2012	33
Kuzma et al. [49]	2008	32
Dolan [48]	2005	30
Dursun et al. [43]	2011	30
Mirelman et al. [63]	2012	26
Sinuany-Stern et al. [64]	1995	23
Til et al. [57]	2014	21
Ahmadi et al. [65]	2015	21
Diaz-Ledezma et al. [47]	2014	17

Considering the set of articles selected in this study, the articles of Baltussen et al. and Goetghebeur et al. [21, 25] were the most cited; presenting as a solution a better allocation of public health resources. The authors developed this study, aiming to benefit disadvantaged groups, making possible the development of a public health policy, as an example: alternatives were presented that support decision making in the treatment of Turner syndrome; Already Lu et al. [26], elaborated a hybrid model for the adoption of new technologies. On the other hand, Doerner et al. [27] has created a combinatorial optimization formulation to choose the best location for a mobile health center.

### Analysis of methodological steps

#### Definition of the decision problem

In addition to the verification of the general data and presentation of the current scenario of the MCDAs in the health area, this study sought to investigate the methodological structure of the included studies in order to identify the techniques and strategies that researchers, specialists and decision makers are agreeing to use for solve a multicriteria problem in the health area.

**Fig. 3:**
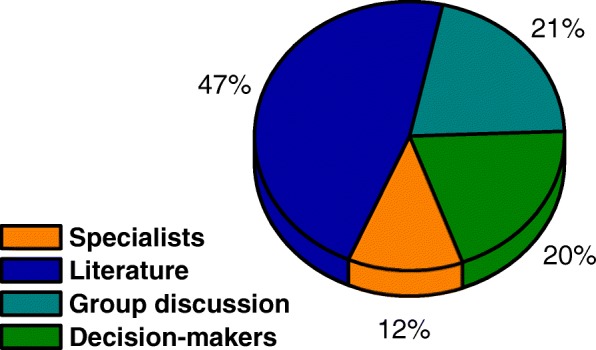
Analysis of the problem definition strategy for structuring the MCDA

To assist researchers and health professionals, this research investigated in the articles included, the following methodological steps: How was the problem established; How did the definition of the criteria occur; The model would be to solve which problem and which weighting method was used. Initially we verified the form that the decision problem was based (Fig. 3).

From a total of 66 articles analyzed, it is visualized in Fig. 3 that 47% of the articles defined the decision problem based on the literature. This represents 31 articles. However, it is important to explain that of these 31 articles, 2 studies added to the literature also consulted experts.

The definition of the problem through discussion groups, formed by interdisciplinary teams, represented 21% of the articles included. This is equivalent to 14 surveys, among these 2 articles besides the discussion group consulted the literature, another 5 in the group discourses asked for support from specialists.

The strategy to define the problem through the knowledge and experience of DMs was attributed to 13 articles analyzed (Fig. 3), which represented 20% of the included studies. Finally, 12% of the analyzed articles used the strategy to define the problem by means of experts, and in 2 articles this was done through a pre-defined questionnaire.

**Fig. 4:**
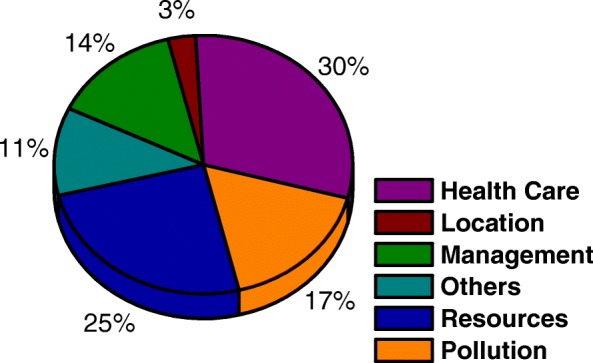
Analysis of the types of intervention that are aided by the MCDA

Within the limits of the decision problem that are defined, the type of intervention that the MCDA will assist is selected, either by sorting, selecting or even ordering the alternatives (Fig. 4). To delimit the decision problem, it becomes necessary to determine the purpose of the model and with it the type of intervention. This identification will have an influence on the final model, considering that the decision process is found in the initial filters [15].

Of the 66 articles analyzed, 30% were concerned with helping health care problems, among them, 7 articles deal with the treatment of diseases, 7 articles on the diagnosis of diseases, 3 articles on disease prioritization, and 3 articles on related issues with medicines.

Being that, 25% of the articles, the intervention was to identify resources. The resources dealt with the choice of information technology in 7 articles, maintenance of equipment in 4 articles, allocation of resources in 3 articles, and choice of equipment in 3 other articles.

On the other hand, 17% of the articles studied, DMs decide, through the MCDA, questions related to environmental pollution, with 9 articles addressing the choice of treatment and more adequate disposal of hospital waste and 2 articles addressing water pollution.

Besides that, 14% of the articles studied, it was evidenced the use of MCDA to support management decisions, with 7 articles addressing the theme in hospital management planning, 2 articles addressed the theme in budget prioritization. Another 2 articles, representing 3% of the total, addressed the theme for choosing the best location for the installation of health facilities.

The Others class is the combination of the works that did not fit into the classes previously explained, deals with interventions related to the sorting of the best doctor for the family, the best teaching hospital, the identification of the risk levels in an emergency department, the criteria equity and efficiency of a health service, to verify that political values in health reflect the values of the population and to support the identification of highly disadvantaged areas, each occurring in each case.

#### Definition of decision criteria

The evaluation model, which conveys the results of the analysis of the consequences of an alternative, is usually too complex to be used directly in decision aid. Instead, one or more criteria should be developed to synthesize the relevant consequences, and be appropriate for the analysis of potential and deep comparisons between them [17]. Thus, knowing how DMs are deciding to set the criteria for decision problems becomes important.

**Fig. 5:**
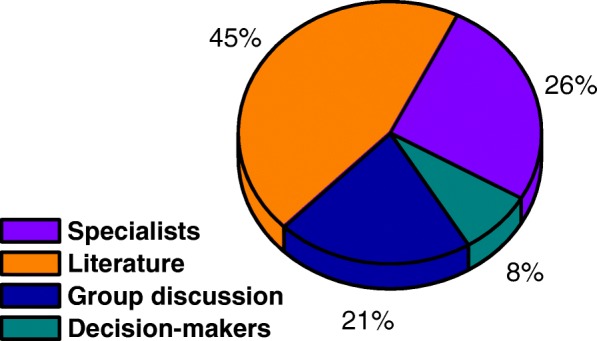
Analysis of the strategy to define the criteria for structuring the MCDA

The criteria used for decision found in the articles studied are shown in Fig. 5.

In 30 of the 66 articles studied in total (45%) defined the criteria as basis in the literature. Of these articles, 23 used only the literature to define the criteria, 5 articles in addition to the literature also had support from experts and two other studies were supported by the literature together the DMs and people interested in the topic addressed by interviews.

The definition of criteria using only specialists was reported in 26% of the evaluated articles. In another hand the use of only discussion groups represented 21% of the total articles analyzed

At the criterion definition stage, only 8% of the papers had DMs as the main decision maker, this represents five articles.

#### Some examples

Examining the way in which health interventions are structured can help in formulating objectives and determining the methodological steps of future work. Here some examples will be presented.

By whom, and how were the limits of the decision problems in the health area defined?

**Decision makers** - They presented the alternatives and parameters needed to structure the decision problem [21, 28];

**Group decision** - All participants gave the written consent form if they were willing to participate in the study. Two focus group discussions were conducted at a six-month interval. Recalling that the concerned parties had different origins [29];

**Literature** - The alternatives were structured according to MARKOV chains for medical gases and vacuum subsystems [30];

**Specialist** - the delimitation was the result of discussions with a specialist. The specialist assesses the needs and according to their knowledge and experience delimits the decision problem, formulating the objective and creating the alternatives [31, 32].

By whom, and how were the criteria identified?

**Decision makers** - The authors defined the four criteria to determine optimal patient management [33];

**Group Decision** - In order to generate an explicit model that helps stakeholders to reflect and analyze relevant issues more clearly, a facilitator is used who works impartially and helps those actors. As participants begin to work together, weighing the criteria and marking the topics, it is proven that this helps those involved to think and generate a comprehension and understanding of the problems, in a shared way [34];

**Literature** - Researching in the literature it is possible to verify that the economic, technical, environmental, and social criteria are elements used as selection criteria, in the process of evaluation of treatment alternatives. However, as there is a need to carry out a comprehensive evaluation regarding treatment alternatives, several authors point out that considering subcriteria related to the above mentioned criteria is an excellent way to perform these evaluations [35];

**Specialists** - The seven associated criteria and subcriteria have been adapted from the NHSIII to fit the United Arab Emirates public health system in the light of discussions with industry experts [36].

#### Types of problem and multicriteria method

To reach the proposed objective, the results referring to the type of intervention and multicriteria method are analyzed. The rationale for this research lies in the importance of the type of problematic to reflect on, the types of results that the analyst intends to achieve, how he sees himself in the process to help achieve those results and how he envisions his recommendations [17]. As well as the knowledge of which multi-criteria methods are most used, which aggregate the data into individual criteria to provide indicators of the overall performance of the alternatives [21].

**Fig. 6:**
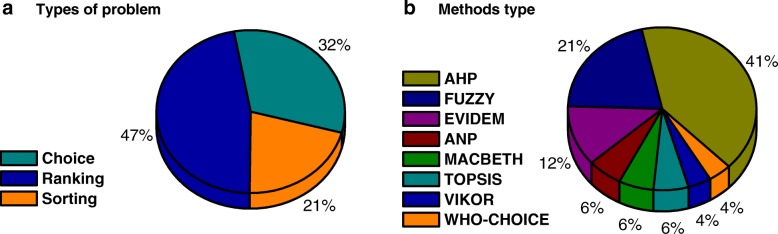
Analysis of the problem and the main MCDAs methods used to aid decisions. **a** Types of problem. **b** Methods type

As shown in Fig. 6a, the type of problematic of 47% of the included articles used the problematic of ranking, this represented 31 studies. The problem of choice was identified in 32% of the articles evaluated (21 articles) and the problem of sorting was finalized with 14 articles, or 21% of the total articles evaluated.

The analysis of multicriteria methods presents only those with more than one application, that is, those that were used in more than one article, so Fig. 6b considers a total of 49 studies. AHP is the most representative method because it was used in 20 articles, representing almost 41% of the total. Following the FUZZY Logic, it represented 21% of the analyzed articles, followed by EVIDEM, ANP, MACBETH and TOPSIS (3 articles each), VIKOR and WHO-CHOICE are present in two studies each.

**Fig. 7:**
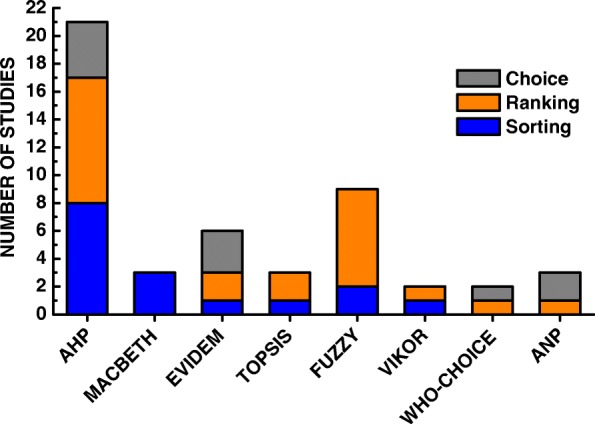
Analysis of the MCDAs methods used to aid the decision of the problems of choice, ranking and sorting

Figure 7 shows the type of problem versus the multicriteria method of included studies. Their analysis aims to visualize what methods researchers are choosing to help solve their decision problems. It is possible to observe that the eight highest occurrence methods of included studies are used in ranking problems. Also, that the AHP, the FUZZY Logic and the EVIDEM are used in the three different problems; TOPPIS and VIKOR in the issues of choice and ranking; The ANP and WHO-COHICE in the problematic of ranking and sorting; And the MACBETH in problems of choice.

In this section, the most representative information was presented, the characteristics of the other studies, as well as those presented in this text, are described in Table 1. These studies were the data used to present the panorama of MCDA publications in the highlighting its main characteristics and methodological steps.

### Exclusions

**Fig. 8:**
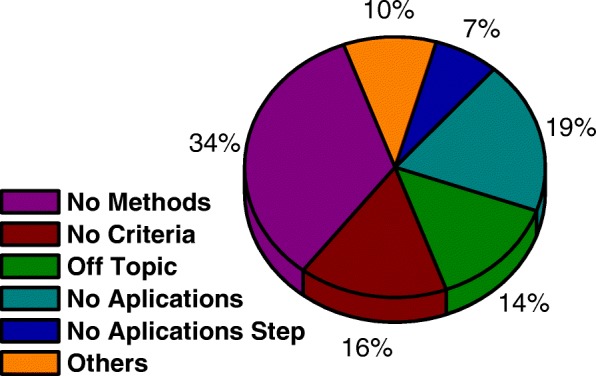
Analysis of the number of articles excluded to reveal the main reasons for the exclusions

The selected articles were analyzed under evaluation of the general questions and under specific questions focused on the methodological structure of the same. As for articles not approved in the screening process, which represented 70% of all articles read completely, were classified according to the types of irregularities found: no method, no criteria, not applied, out of subject, no application steps, and others (Fig. 8).

The criteria for exclusion of the articles of the present study were cataloged, with 34% of articles not having methods described in the text or not using MCDA. In 19% of the articles excluded from the present study, there were no real applications, with only a descriptive of the problems to be treated. In 16% of the articles excluded, the criteria were not identified or were not explicitly stated. In these articles, the decision matrix were not developed, and in some cases, the description of the criteria does not exist.

Some studies analyzed fled the central theme, these being represented by 14% of the articles excluded. In these cases, the subjects did not involve the health area or, moreover, did not use the MCDA in general, 10% of the articles excluded from the present study were due to several other categories of exclusion presented in the methodology of this work. These categories include animal research and reviews that have gone unnoticed by screening. Finally, it was found that 7% of excluded articles did not follow the methodological structure with a whole.

In relation to the articles selected for study (30% of articles read), in the scope of the general questions, the growth rate of the number of publications, the countries where they were applied, the author of the articles and the periodicals where they were published were evaluated. And from these data it was possible to determine not only a significant growth rate in the number of structured studies, but also the dissemination relation of this methodological structure of application.

Observing the lack of dominance in the aspects of the main authors and in the application sites, it can be assumed that the knowledge of the MCDA is undergoing an expansion in the range of health care, in other words, knowledge of the structured application of multicriteria is spreading and being applied and dominated by a wider range of scholars.

In the eligibility stage, 239 articles were included and only 66 selected. The analysis of exclusion reasons revealed that 21% of the articles do not make clear in their research the main methodological steps of the MCDA. And it is possible to apply the MCDA to help decision making in the health area, even without using a multicriteria method, but a purely mathematical model belonging to other areas of knowledge, such as Statistics [37].

## Discussion

Compared to the figures found in Fig. 2a, we are able to affirm that between the 1990s and 2000, the growth of structured studies with a structured methodology was 450% (1990 − 1999 = 2 publications, 2000 − 2009 = 9 publications); (2000 − 2009 = 9 publications, 2010 − 2017 = 55 publications), with an increase of 611% Table 4.

**Table 4:** Growth analysis of the studies to indicate the growth of the structured articles with the structured methodology

Decade	Quantity	Growth
1990	2	-
2000	9	450%
2010	55	611%

These data clearly indicate the growth of this type of study and the great importance that these researches have for the academic population over time. It also emphasizes the growing use of a structured methodology in the researches involving the subject, which strengthens and disseminates the MCDA.

In relation to the countries that most perform publications, Fig. 2b, we have a small dominance, since we have Canada [25, 29, 38–41]. Turkey [8, 31, 42–45] and USA [33, 46–50] with six publications each, followed by China [10, 51–54] and the Netherlands [21, 55–58] with five publications each, and Spain [28, 30, 59, 60] with four publications, with six countries accounting for 48% (thirty-two studies) of all publications by all thirty-three study countries.

On Fig. 2c, it can be said that the studies had fifty-eight different first authors, and that the group of seven authors shown in chart c, refers only to 23% of all publications (15 studies), while others Fifty-one authors with only one published article represent 77% of the research; thus affirming that there is no dominance of authors in the scenario studied.

In relation to the analysis of Fig. 2d, it can be seen that the nine journals analyzed in the graph represent a 30% share of all studies (20 articles), but since we have fifty-four journals with publications, this significant value can not be considered of dominance, and a homogeneous distribution is represented for the reference journals in the area.

In addition to the general data, issues related to the structuring and application of the multicriteria methodology were also analyzed. In Fig. 3 a small dominance is identified in the studies that use the literary questions to determine the research problem. In Fig. 4, the research objective is analyzed, it is clear that there is no dominance, but a highlight for two points, first research on the treatment of diseases (30% of research) and allocation of resources (25%). Figure 5 shows the methodology used to determine the criteria, and in this case, presented dominance of the literature (45%). Figure 6a shows the division of the problems presented in the articles, highlighting those classified as ranking (47%) and Fig. 6b shows the main methods used, especially AHP (41%) and Fuzzy Logic (21%). And Fig. 7 shows the relationship between the two previous data, identifying the correlation between the problem and the method used.

In addition, it is worth noting that in the literature investigated there are some trends and challenges that should be considered by those applying MCDA in health care. First, the criterion definition stage tends to happen more in tandem than the structuring of the problem. In the articles investigated, the percentage for all classes remains balanced, but when compared to the Decision-Makers class, it appears more in the structuring of the problem, but when it goes into the criteria definition phase, the tendency is for the process to occur participatory manner.

Second, even though it does not appear, data analysis is important, highlighting the role of the analyst, responsible for managing the entire decision process. Third, from the structuring and definition of the criteria of the decision problem, the analyst already has enough information to choose the best, or better methods for the construction of the multicriteria decision model. Fourth, in the studies analyzed there is a tendency for participatory processes. Fifth, the review shows that the greatest interest of all who use the MCDA to aid their decisions is after the resolution to visualize their alternatives as a ranking, proving one of the advantages of the MCDA, which is, in addition to saying what to do, it shows you how to do it.

This research was limited in two areas, purposely when the level of the journals used and the selection of research platforms were selected, and the acceptance of the only peer-reviewed articles; but also in a disproportionate way by the visualization of the articles, since some of the articles researched were not open.

## Conclusion

The application of the MCDA methodology has been disseminated and applied around the world, and increasingly used in widely humanized areas such as health. The studies presented in this review are shown as indicators of the current scenario, exposing not only the importance of the MCDA, but also its methodological structure of application.

Regarding the general aspects of the studies, a growing trend was observed in the application of these methods, in addition to having no dominance in relation to the authors of the publication and the periodicals where they are published, but some countries stand out in terms of the number of published researches, such as such as Canada, Turkey and the USA.

In the definition of the problem of decision and stage of definition of the criteria, of studies included the literature presented the greatest demand for those who wish to structure their decision problem, however, it was verified that the literature added to group discursions showed good acceptance. Finally, it was verified by the analysis of the problematic, that the MCDA to solve problematic of Ranking has extensive application in the health area. As for the methods, the dominance of the AHP and FUZZY Logic was remarkable.

With this, it is possible to observe, through the data of this review, that more than the multicriteria methods, the multicriteria decision model has been highlighted, also in the health area. In addition, the study can guide new applications and techniques using MCDA in the health care.

For future work, the possibility of included studies focuses on descriptive researches, where mathematical methods are not used, aiming at the methodological application of the MCDA.
